# Boundary politics and the social imaginary for sustainable food systems

**DOI:** 10.1007/s10460-021-10214-0

**Published:** 2021-05-02

**Authors:** Kim L. Niewolny

**Affiliations:** Virginia Tech, 282 Litton-Reaves Hall (0343), Blacksburg, VA 24061 USA

**Keywords:** Sustainable food systems, Social movements, Anti-racism, Social imaginary

## Abstract

In this essay, Kim Niewolny, current President of AFHVS, responds to the 2020 AFHVS Presidential Address given by Molly Anderson. Niewolny is encouraged by Anderson’s message of moving “beyond the boundaries” by focusing our gaze on the insurmountable un-sustainability of the globalized food system. Anderson recommends three ways forward to address current challenges. Niewolny argues that building solidarity with social justice movements and engendering anti-racist praxis take precedence. This work includes but is not limited to dismantling the predominance of neoliberal-fueled technocratic productivism in agricultural science and policy while firmly centering civil society collective action and human rights frameworks as our guiding imaginary for racial, gender, environmental, and climate justice possibilities for sustainable food systems praxis. She concludes by exploring the epistemic assertion to push beyond our professional and political imaginaries to build a more fair, just, and humanizing food system.

I begin this essay by sharing my appreciation for Dr. Molly Anderson’s 2020 AFHVS Presidential Address. Rightfully, her message of moving “beyond the boundaries” focuses our gaze on the insurmountable un-sustainability of our globalized food system. As it has been said many times before, the disaffection with the material and bodily inequities of our “maddening” industrialized food system is increasingly difficult to deny (Orlie 2009). Yet the current system persists through a complex set of power relations that continue to disrupt our social, ecological, and cultural lives from the local to the global. For Anderson, these disruptions are many that require greater coordination and action on the global stage if sustainable food system transformation is to take place. Several challenges lie ahead for this work that stretches across our academic, policy, and grassroot spaces of theory and practice. In agreement with Anderson, I argue that these challenges are manifested in our professional and political imaginaries that enable some possibilities real while limiting others. In order to transform the food system, we must transgress the boundaries that comprise normative and oppressive knowledges, practices, and structures. Similarly, I argue these challenges include dismantling the predominance of neoliberal-fueled technocratic productivism in agricultural science and policy while firmly centering civil society collective action and human rights frameworks as our guiding imaginary for racial, gender, environmental, and climate justice possibilities for sustainable food systems praxis.

Going further, we must also acknowledge how issues of power and privilege are heightened to new levels of severity as the COVID-19 pandemic deepens the gap of food access and availability injustice. How to address the current moment and future crises through an intersectional justice and human rights lens is what requires our immediate attention. A pandemic-informed report published by the International Panel of Experts (IPES) on Sustainable Food Systems in April 2020 is a stark reminder that the time for serious discussions and honest assessments of what is and is not working in the food system is long overdue. Yet a year after the outbreak took hold, concerns about the food supply chain continue to make headlines pointing out the many vulnerabilities in the system. Large economies of scale and vertically integrated production systems are still too rigid to nimbly move, leaving many farmers with hardships and difficult decisions for their farms and communities. At the same time, the emergency food system in the U.S. continues to be strained in demanding ways as individuals and families struggle with limited incomes, affordable housing, healthcare costs, and caregiving responsibilities, disproportionally impacting women and communities of color. We also see increasing concerns for the health and safety of frontline food and farm workers as they seek to make a livelihood in kitchens, meat processing plants, and crop fields with few safeguards or fair wages for their “essential” labor. Taken further, these concerns for protecting the most vulnerable during the pandemic remain largely unheard by those in positions of power on the national and global front despite grassroot mobilization. As people have pointed out for decades, our domestic and global food economy was established on the labor of enslaved people and the foundation of stolen Indigenous land. The current moment reveals how these legacies of oppression linger as the systematic exploitation of Brown and Black bodies continues in order to reach the goals of productivity and efficiency through domestic and transnational capital markets.

This unfathomable health event has indeed exposed weaknesses, challenges, disparities, and underlying inequities in the U.S. and global food system with little to no accounting for environmental justice, health equity, and human rights. As an academic with teaching, research, and extension responsibilities in a U.S land-grant university, I feel these and other points of vulnerability and crisis are not nearly stressed enough in our teaching, research, and outreach efforts. The opportunity to better understand and address the food system as a dynamic system that takes on a number of issues, politics, and priorities has never been more significant. Of importance, this opportunity requires us to embrace Anderson’s claim of reexamining and resisting the current emphasis on measures of efficiency and productivity. It also means illustrating and dismantling how these discourses and practices intersect with neoliberalism, settler colonialism, white supremacy, and patriarchal frames and actions. For example, the privileging of neoliberal discourse with technocratic and productivist measures in the food system is far from new. A focus on efficiency alone reifies a strong tendency to reduce, flatten, and distill the complexities of our food system from soil to plate down to a specific suite of linear and technocratic solutions and practices (Pimbert 2018; Alkon and Guthman). Going further, the reification of neoliberal efficiency and technocratic problem solving in agriculture fails to bring to light the interdependence and depth of our socio-eco relations or the larger political ecology of food and agriculture (Galt 2016). Mary Hendrickson, Philip Howard, Emily Miller, and Doug Constance take this point further in their November 2020 report to the Family Farm Action Alliance titled, “The Food System: Concentration and its Impacts.” They caution that the current agricultural trajectory, with its overemphasis on measures of efficiency and productivity, callously operates to concreate power in the hands of a few, further obfuscating the material and discursive possibilities to re-imagine our global food system as one where all may benefit and thrive:

“The distribution of power in the food system, embodied in the power to make decisions about what food is produced, how, where and by whom, as well as who gets to eat—and what they get to eat, is our major focus of concern because of the negative impacts of those decisions to farmers, workers, communities and our ecology. Without a rebalancing of economic and political power within the global food system, humanity confronts a crisis over our very sustenance (p 2).

Food scholars and activists continue to grapple with these these and other social change critiques and possibilities. Reflecting on the current moment allows us to reengage with such critical questions that have been asked many times before: What does a humane, resilient, and fair system actually look like? How do we build equity in the fabric of our food system today and for future generations? How do we move toward a restorative food future? What strategies of resistance, disruption, prevention, and repair make sense? For Anderson, the answers to some of these questions lie in places that are beyond our mainstream ways of knowing and acting. We need to explore more deeply, widely, and compassionately to learn how people near and far are meeting their food system challenges. In this spirit, she recommends three strategies to push the boundaries “that are holding back progress” on addressing some our greatest food system issues. These approaches include: 1) engaging more intentionally with international opportunities and colleagues from governmental to civil society organizations to refine our critiques of the globalized food system, 2) interacting and organizing with global social movements to build new alternatives, and 3) committing to anti-racist work to seek a deeper understanding of the way structural and institutional racism in our food system operates while also developing a pathway to action to help dismantle the attitudes, practices, and structures that hold racism in place. While each of these opportunities may look a little different from each other, they equally demand we engage with an astute critique of the current conditions to better problem solve. They also require an examination of our professional motivations and trajectories so that “boundary work” is more possible. Anderson also recommends we take the meaning of solidarity seriously by illustrating greater commitments to social movement actors and communities who first-hand experience systems of oppression and violence.

Of these opportunities, I argue that building solidarity with social justice movements and engendering anti-racist praxis take precedence. In doing both, our priorities could shift toward critical research, education, and policy changes with a focus on uplifting the needs and concerns of those most vulnerable to the disruptions and inequities in the food system. This would also demand a redistribution of resources and decision-making power to communities most impacted by the historical and hegemonic race-gender-class politics of food access and availability. In this effort, it will prove crucial that those who benefit from pervasive systems of privilege (especially White privilege) to listen to and act in solidarity with civil society movements in the Global South and Global North who are actively re-imagining land, food, and environmental justice and liberation through such frameworks as agroecology, food sovereignty, and collective agency (see Agyeman and Alkon 2011; Daigle 2017; Holt Giménez et al. 2017; Penniman 2018; White 2018). It is in this vein that Anderson’s Presidential Address sharpens our awareness of a groundswell of academic and grassroot activism currently addressing the social, economic, and ecological un-sustainability of the globalized food system.

Going further, I argue that localized and regional movements spaces—wherever you live and work—are important and necessary to build and foster. This is not to say we should not reach out to the global community to build alliances and networks for enhanced learning and social action for common causes as Anderson suggests. Instead, it is critical to remember that social justice movements and collective action efforts may differ in scale and scope yet together hold promise in amplifying food system change work from all parts of the world. It is therefore important that food system scholars and policy makers work to heighten the activities and possibilities of frontline communities and movement actors in the large *and* small spaces and among all the parts of the system to transgress normative and oppressive boundaries. Learning to “do” this work requires us to “see” the everyday experiences of struggle and oppression that intersect with the complexity of our food system politics. This includes but is not limited to recognizing food system complexities and intersections to catalyze: 1) agroecological possibilities in research, education, and extension agendas; 2) healthy and equitable food access policy; 3) food and farm system worker protections; and 4) the emancipatory potential of food sovereignty praxis. Consequently, those working toward food system change need to amplify the systems work of organizations led by Black, Indigenous, and people of color who are already advancing just and equitable food systems globally, regionally, and locally. At the local level, our Virginia Tech Center for Food Systems and Community Transformation is grateful for the opportunity to build relationships with food justice leaders and organizations in Virginia and beyond whose community food work is a rich seedbed for social critique, knowledge creation, and social action nested in anti-racist and restorative justice frameworks. In this vein, I also agree with Andersson that we should connect with non-food movements who uphold social justice values. I would add that such movements should be rooted in anti-racist and decolonizing praxis to dismantle settler colonialism and systems of White supremacy. It also means engaging in activism for gender equity and LGBTQ + rights. For me in the Appalachian and rural regions of the U.S south., this has meant working with and learning from organizations and coalitions whose goals are to address anti-Black racism, Indigenous sovereignty, fossil-fuel pipeline construction, climate justice, collective economy alternatives, and mutual aid initiatives. Like many others, I have learned that multi-sector collaborations and networks open new possibilities for just and systemic social change where food justice intersects.

What I found most satisfying about Anderson’s calls for action is the underlying assertion that food scholars and change makers need to push beyond our professional and political imaginaries to help build a more fair and humanizing system. I argue that in order to circumvent current barriers, there is a need to more deliberately “unthink” the orthodoxies that govern our ideas of the possible. That is to say, we must do more than find news way to theorize and critique the forces of change in our unjust food system. We also need to move away from linear, technical “best practice” solutions and fixed notions of foods system work and knowledge production. In this way, I am encouraged by the generative possibilities of community food work as political praxis. This includes embracing food systems work as more reflexive, emergent, and dialogical—that is, less extractive. More specifically, there is much to learn by uplifting the creativity of multi-sector and multi-racial coalitions who are at the forefront of alternative transformations through a more dynamic and collaborative structure and vision for food justice (e.g., HEAL Food Alliance). By challenging our boundary politics in these ways, we might then ask: What worlds are being made through our practices, and more importantly, how could they be done differently for just ends?

In conclusion, Anderson’s address gives us guidance to keep moving forward despite the challenges of our globalized food system. While we may feel disjointed and disrupted, I am reminded of the necessity of hope in generating new roots and new growth in times of despair (Lear 2008). To push boundaries requires more than doing things differently, it requires us to “see” the world differently. Drawing upon the work of bell hooks (2003), we have an immense opportunity before us, through an empathetic and critical praxis, to help devise and enact a new social imaginary that moves us towards a more equitable food system. As I have said elsewhere, I am hopeful that our current moment enables us with new capacity and courage to bring about the life-affirming possibilities we seek. The extent of these possibilities go as far as our imaginations can take them (Niewolny 2020).
